# Primary small‐cell carcinoma in the lung was found after the cold snare polypectomy of the small metastatic lesion in the cecum: A case report

**DOI:** 10.1002/deo2.266

**Published:** 2023-06-26

**Authors:** Daisuke Suto, Masashi Yoshida, Hidehiko Yamada, Takayuki Akita, Yosuke Osawa, Kiichi Sato, Takaaki Otake, Yoshimasa Nakazato, Nobuhito Arakawa, Hironori Ohdaira, Yutaka Suzuki, Yutaka Kohgo

**Affiliations:** ^1^ Department of Gastroenterology International University of Health and Welfare Hospital Tochigi Japan; ^2^ Department of Surgery International University of Health and Welfare Hospital Tochigi Japan; ^3^ Department of Pathology International University of Health and Welfare Hospital Tochigi Japan; ^4^ Department of Respiratory International University of Health and Welfare Hospital Tochigi Japan

**Keywords:** cold snare polypectomy, colorectal metastasis, non‐hyperplastic polyps, small-cell lung cancer, thyroid transcription factor‐1

## Abstract

Metastasis from small‐cell lung cancer to the colon is very rare. A 74‐year‐old man without respiratory or abdominal symptoms underwent a follow‐up lower gastrointestinal endoscopy after a polypectomy. He was diagnosed with a 5 mm IIa non‐hyperplastic polyp in the cecum and underwent a cold snare polypectomy. The histopathological findings confirmed the diagnosis of small cell carcinoma. The tumor was positive in the deep margins of the submucosal layer. Subsequent systemic examination revealed a mass in the lower lobe of the left lung. Thus, the tumor in the cecum was determined to be a colorectal metastasis from primary small‐cell carcinoma of the lung. Metastasis to the colon was diagnosed as small‐cell lung cancer based on local positivity for thyroid transcription factor‐1 and morphologic and immunochemical features. To our best knowledge, this is the first report of colon metastasis from small cell carcinoma identified by endoscopic treatment.

## INTRODUCTION

Lung cancer is a leading cause of cancer‐related deaths worldwide.^1^ Colonic metastasis from small‐cell lung cancer (SCLC) is rare. Moreover, most abdominal metastases from lung cancer are squamous cell carcinomas found in the liver, pancreas, and spleen.^2^ Among them, the gastrointestinal tract is not a common site of lung cancer metastasis and is often underdiagnosed during clinical follow‐up in patients with lung cancer.^3^ Regarding small cell carcinoma, although we looked for “cold snare polypectomy” (CSP), “hot biopsy”, “endoscopic mucosal resection”, and “endoscopic submucosal dissection” on PubMed, no reference was found to date. Hence, this is the first report of colon metastasis from small cell carcinoma identified by endoscopic treatment.

Due to its asymptomatic progression, gastrointestinal metastases are often diagnosed at advanced stages, leading to an extremely poor prognosis. In this case, polypectomy was performed for the initial diagnosis of primary SCLC. We report this case to emphasize the potential beneficial value of this diagnostic process for informing future clinical practice to improve patient outcomes.

## CASE REPORT

**FIGURE 1:**
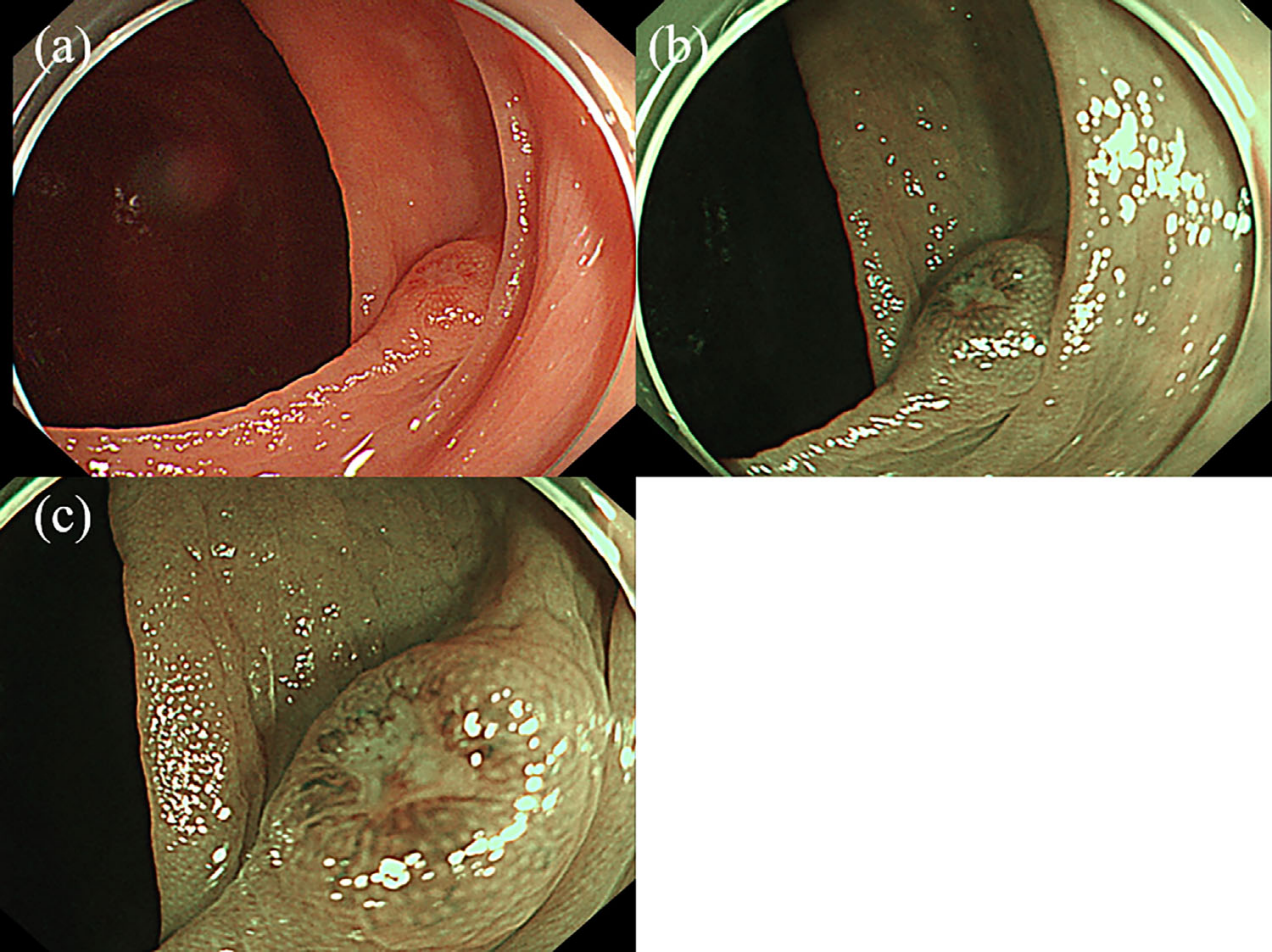
Endoscopic image of a 5 mm‐sized polyp of the cecum. (a) Erythematous depressed polyp in the cecum. (b) Narrow‐band imaging images of the polyp. (c) Narrow‐band imaging magnified image of the polyp.

**FIGURE 2:**
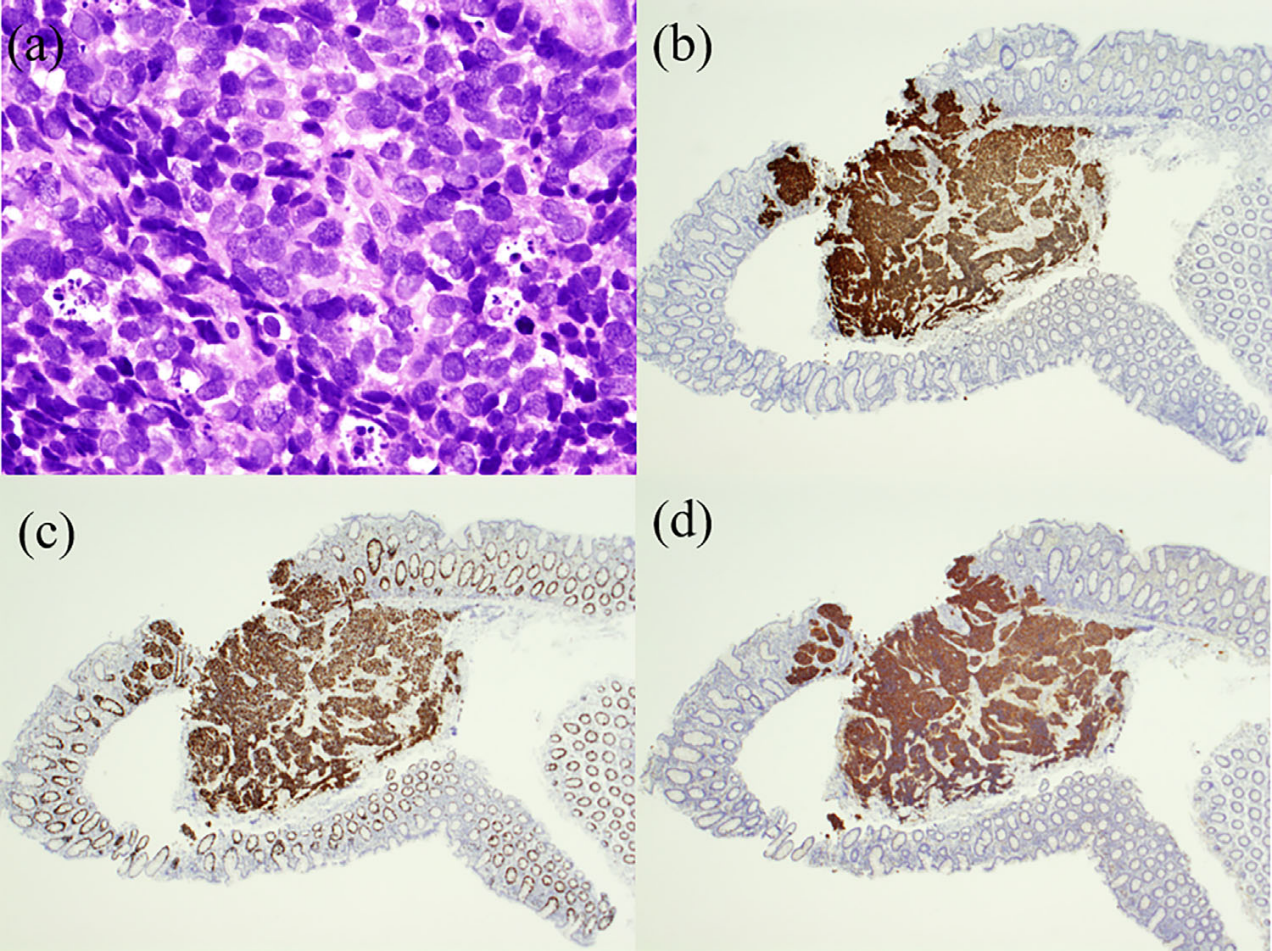
Hematoxylin‐eosin staining and immunohistochemistry of cold snare polypectomy specimens. (a) Hematoxylin‐eosin staining of cold snare polypectomy specimens (×400). (b) Tumor cells positive for thyroid transcription factor‐1 immunostaining (×40). (c) Tumor cells positive for Mib‐1 immunostaining (×40). (d) Tumor cells positive for synaptophysin immunostaining (×40).

**FIGURE 3:**
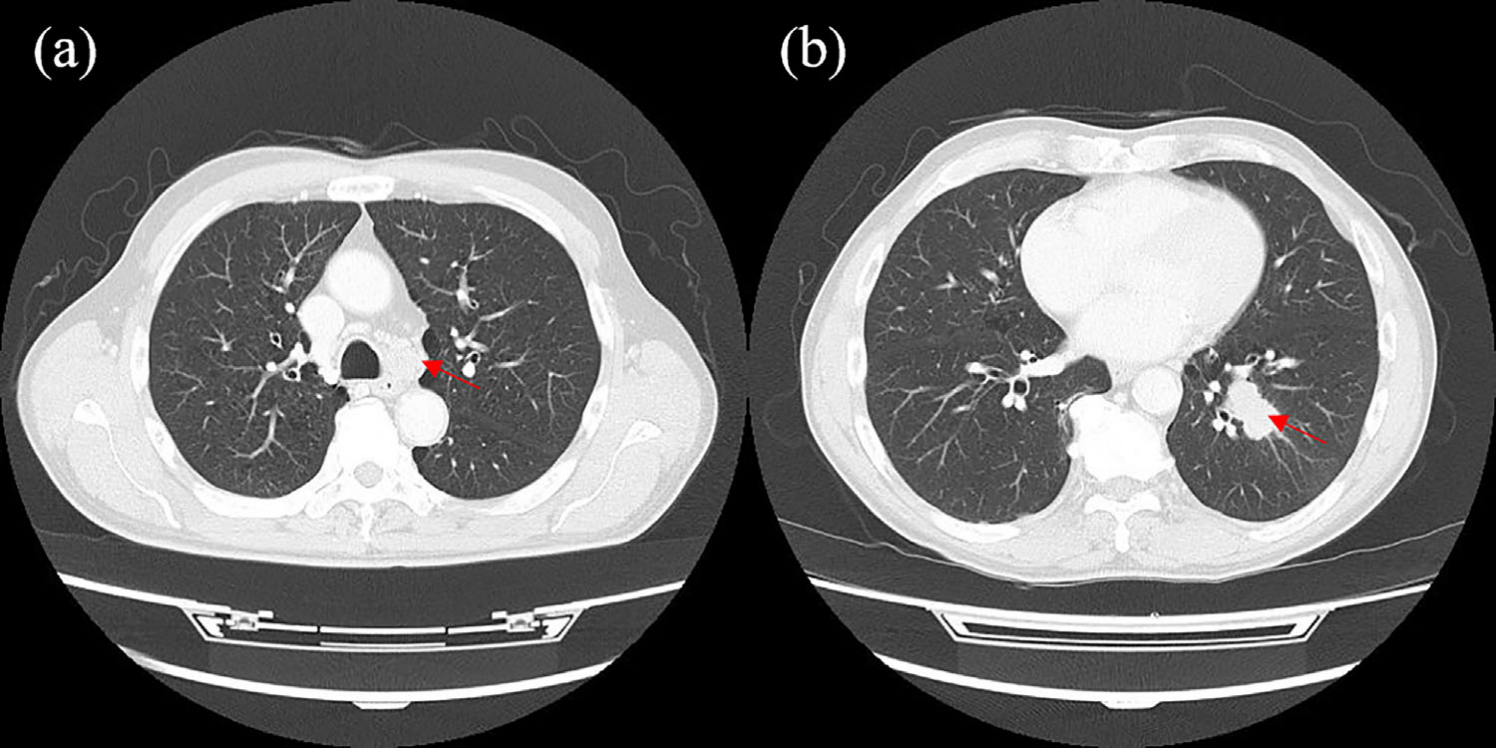
Whole body computed tomography scan. (a) A computed tomography scan shows an ill‐defined mass in the left lower lobe (red arrow). (b) Enlargement of the left hilar lymph nodes (red arrow).

**FIGURE 4:**
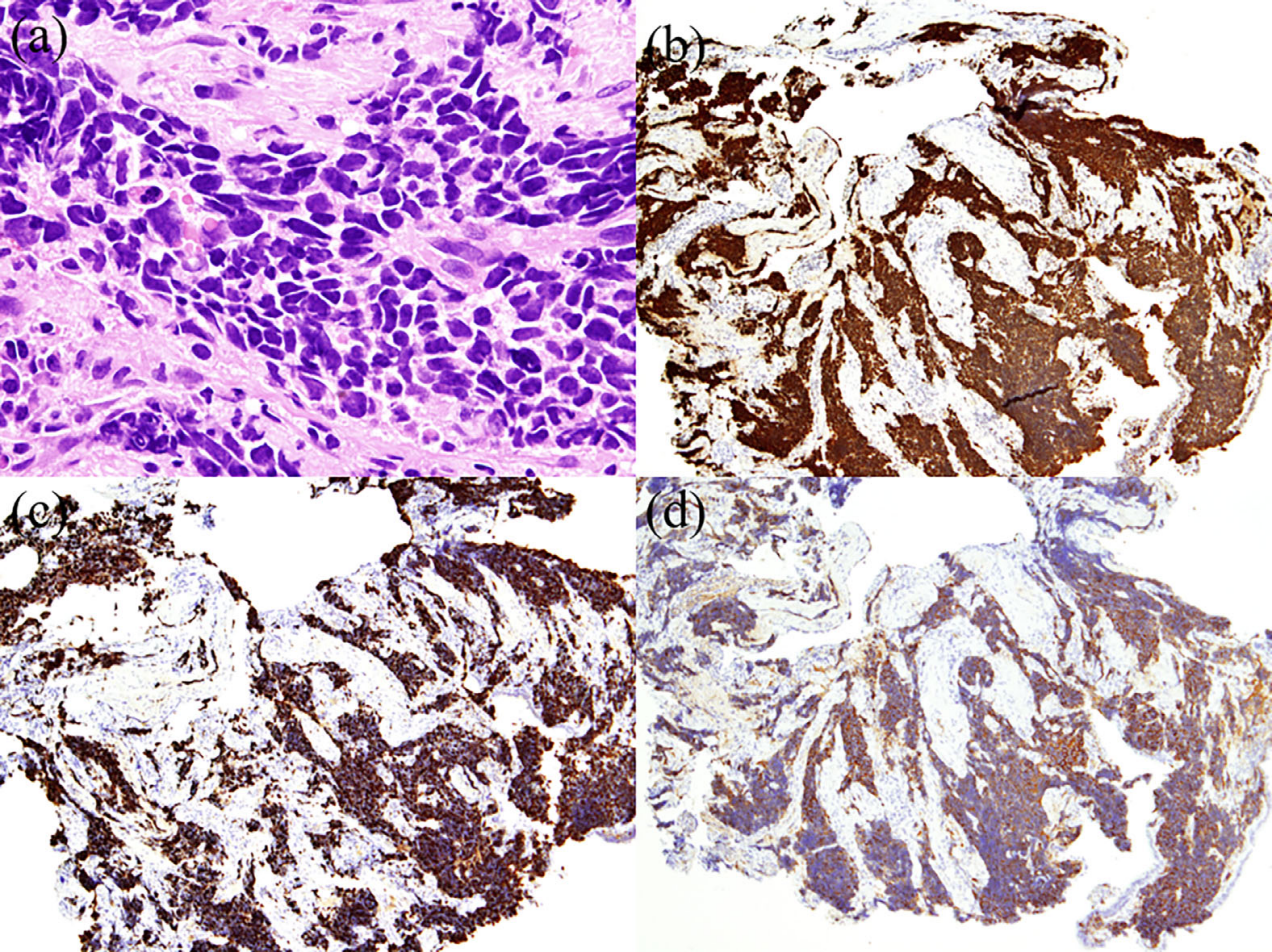
Hematoxylin‐eosin‐stained and immunohistochemistry specimen for the tumor in the lower lobe of the left lung. (a) Hematoxylin‐eosin staining of lung biopsy tissue (×400). (b) Tumor cells positive for thyroid transcription factor‐1 immunostaining (×40). (c) Tumor cells positive for Mib‐1 immunostaining (×40). (d) Tumor cells positive for synaptophysin immunostaining (×40).

A 74‐year‐old male patient underwent a follow‐up lower gastrointestinal endoscopy after polypectomy. As a result, a 5 mm IIa polyp (Figure 1a) was found in the cecum, which had not been identified a year earlier. The findings of narrow‐band imaging (Figure 1b,c) were different from these of adenoma. However, the white depressed area without obvious irregularity of the surface structure with slightly irregular margins set it apart from a hyperplastic polyp. CSP was performed and subsequent histopathological examination confirmed the diagnosis of small cell carcinoma. Hematoxylin and eosin‐stained magnified images showed substantial proliferation of basophilic tumor‐like cells extending to the mucosal fasciculus (Figure 2a). Immunohistological analysis revealed that the lesion was positive for thyroid transcription factor‐1 (TTF‐1; Figure 2b), Mib‐1(Figure 2c), and synaptophysin (Figure 2d). The margins of the resected tissues were positive vertically in the submucosal layer. Therefore, it was diagnosed as a small cell carcinoma in the mucosa. A whole‐body computed tomography scan revealed a 35‐mm tumor in the lower lobe of the left lung, enlarged hilar lymph nodes, and lymph node metastasis in the tracheal bifurcation to the right adrenal gland and sacrum, indicating colorectal metastasis from the primary SCLC (Figure 3a,b). Subsequent bronchoscopy led to a biopsy of the left lung mass, of which hematoxylin and eosin staining revealed a bare nucleated dysplastic enhancing tumor resembling a small cell carcinoma (Figure 4a).

Immunohistological analysis of the left lung tumor biopsy specimen also revealed positive results for TTF‐1 (Figure 4b), Mib‐1 (Figure 4c), and synaptophysin (Figure 4d). The histopathological diagnosis was cecal metastasis from SCLC since positivity for Mib1 was more than 90% and staining for synaptophysin and TTF‐1 also showed positive results. The patient did not exhibit symptoms such as cough, expectoration, hemoptysis, diarrhea, constipation, or abdominal pain. The patient has been scheduled to receive anticancer therapy with carboplatin, etoposide, and durvalumab for SCLC in the Department of Respiratory Medicine at our hospital.

This study was approved by the Ethics Committee of our hospital (approval number: 23‐B‐8). Written informed consent was obtained from the patient for the publication of this case report and any accompanying images.

## DISCUSSION

This is the first report of colon metastasis from primary small cell carcinoma identified through endoscopic treatment of colorectal polyp. In this case, lower endoscopy was initially performed, followed by CSP to identify a 5 mm polyp that differed from a hyperplastic polyp or adenoma. It was diagnosed as a small cell carcinoma. Additionally, subsequent systemic examination led to the final diagnosis of colon metastasis from primary SCLC.

Endoscopic findings of metastatic colorectal cancer have been reported to be diverse, including colon wall thickening, obstruction due to mucosal swelling, erythema, and erosions. Metastatic colorectal cancers generally manifest with submucosal tumor‐like growths or extraintestinal wall compression as they grow inside or outside the intestinal wall.^4^ However, it has been reported that there are no characteristic endoscopic findings of colon metastases from lung cancer.^5^ In this case, the endoscopic findings showed that the lesion was gently elevated. In addition, narrow‐band imaging of the margins of the lesion revealed regular vascular structures that were useful for differentiating submucosal tumors from other diagnoses. However, no thickening of the colonic mucosa or mucosal swelling was observed. Furthermore, the lesion showed slight erosion in its center, wherein CSP for diagnostic and treatment purposes allowed us to establish a diagnosis of colon metastasis from SCLC. In the future, accumulating cases and evaluating whether the central erosion of the lesion and the presence of regular vascular structures around the erosion could be characteristic of endoscopic findings of colorectal metastasis from SCLC is necessary.

Immunohistochemical techniques are useful for identifying the primary site of metastatic gastrointestinal tumors. Similarly, immunostaining for TTF‐1 has been found to be useful in identifying primary lung cancer.^5^ In this case, immunostaining of the lesion revealed positivity for TTF‐1. TTF‐1 is a 40.2 kD transcription factor member of the NKx2 family and is normally expressed in thyroid and lung epithelial cells.^6^ Although TTF‐1 is expressed in the nuclei of 60%–75% of lung adenocarcinoma cases, it is rarely expressed in gastric and colon adenocarcinomas.^7^ In the present study, a tumor biopsy of the lung was also positive for TTF‐1. Thus it was determined that the metastatic tumor in the gastrointestinal tract had a pulmonary origin.

In this case, a 5 mm polyp was found in the cecum during a follow‐up lower endoscopy after polypectomy. Furthermore, CSP was performed for both diagnosis and treatment. Although the endoscopic images later reviewed showed that narrow‐band imaging of the margins of the raised lesion was a regular vascular structure, clearly different from an adenoma, CSP was performed. According to an American study, endoscopic resection of all adenomatous polyps was effective in preventing colorectal cancer and consequently indicated.^8^ On the other hand, according to the Japanese polyp guidelines, polyps smaller than 5 mm have a low likelihood of cancer and can be monitored through follow‐up examinations.^9^ However, endoscopic resection should be performed of flat and depressed neoplastic lesions even if ≤5 mm in size (recommendation strong [agreement rate, 100%], level of evidence D).^9^ In this case, endoscopic treatment of a 5 mm elevated lesion with a slight depression led to the diagnosis and treatment of lung cancer without delay. If the 5 mm elevated lesion with a slight depression was left unnoticed or followed up, the diagnosis of lung cancer and treatment would have been delayed.

In conclusion, polypectomy was performed for the initial diagnosis of primary SCLC in this case and is preferred for the diagnosis of small non‐hyperplastic polyps.

## CONFLICT OF INTEREST STATEMENT

None.

## ETHICS STATEMENT

All procedures followed have been performed in accordance with the ethical standards laid down in the 1964 Declaration of Helsinki and its later amendments.

## INFORMED CONSENT

The patient has provided written informed consent for the publication of this report.

## Data Availability

All data generated or analyzed during this study are included in this published article.
